# Mechanical failure of total hip arthroplasties and associated risk factors

**DOI:** 10.1007/s00402-022-04353-0

**Published:** 2022-01-28

**Authors:** Henrik C. Bäcker, Chia H. Wu, Arne Kienzle, Carsten Perka, Clemens Gwinner

**Affiliations:** 1 Department of Orthopaedic Surgery and Traumatology, Charité Berlin, University Hospital Berlin, Chariteplatz 1, 10117 Berlin, Germany; 2 Department of Orthopedics and Sports Medicine, Baylor College of Medicine Medical Center, Houston, TX USA

**Keywords:** Arthroplasty, Hip, THA, Failure, Complication, Outcome

## Abstract

**Introduction:**

Mechanical failure of total hip arthroplasties is a rare but devastating complication. With increasing numbers in primary arthroplasty implantation, revision surgeries are indicated more often. Therefore, understanding the mechanism and the location of failure is essential in determining proper treatment. Aim of this study was to identify mechanical failures of all total hip arthroplasties performed in a major academic center as well as the associated risk factors such as BMI and sports.

**Methods:**

A retrospective trial was conducted using our prospective arthroplasty database. Database was searched for all patients presenting with mechanical failures of total hip arthroplasty (THA) to the emergency department between 2011 and 2019. All medical charts and radiographs as well as surgical reports were analyzed to identify demographics, implant choice in addition to location of failure and subsequent treatment.

**Results:**

In total, 13 patients suffering from mechanical total hip implant failure were found. The femoral neck (conus) was broken in four patients, the stem in five cases, one broken inlay, two cup failures and one conus dislocation. The mean BMI was 31.42 ± 5.29 kg/m^2^ including five patients who have obesity class II. In all cases, revision surgeries were indicated. No structural causes or underlying risk factors such as repeated physical load (i.e. in sports) were identified.

**Conclusion:**

Implant failure does not seem to correlate with participation in sports or BMI. Catastrophic failure of implants is a technical challenge requiring special extraction instruments that can be difficult even for experienced surgeons. It should be noted that functional outcome is often worse for this group of patients after surgery than comparing against those revised for loosening.

**Level of evidence.:**

III, Retrospective Trial.

## Introduction

In increase in number of total hip arthroplasties has led to more revision arthroplasties. In the initial years, structural implant failures were described more commonly. However, improvements in surgical techniques and implant quality have led to a decrease in these complications. [1, 2] furthermore, U.S. Food & Drug Administration (FDA) approval is monitoring these events closely in order to minimize adverse events.

Biomechanical studies showed that critical stress within the thin titanium dioxide (TiO_2_) can lead to micro cracks that subsequently become structural failure [3]. In addition, corrosive environment such as oxidative stress may predispose implant to failure. [4] As such, modular prosthesis is thought to be at higher risk for failure especially at the taper junction. [5] These have shown increased fretting corrosion at the modular interface [6–8]. Some have postulated that there is a link between body weight, sports and implant failure, such that some manufactures actually warn of a higher risk of implant failure in patients with BMI greater than 35 kg/m^2^. To this date, no bodyweight limitation is mentioned in the FDA approval. In addition, scratching, notching or striking the prosthesis is described to compromise the implant’s load-bearing capacity. [9] In literature, no correlation between participation in sports and implant failure has been found. [10, 11]

In the literature, modular revision hip arthroplasty failure rate from implant fracture is reported to be 0.30% (*n* = 113/37,600). It occurs most commonly at the modular junction or close to the additional neck segment in 79% of the cases. Hereby, especially improper use of the implant was described with 0.11%. On the other hand, only one case for primary implant failure was reported. [12]

Purpose of this study was to investigate the occurrence of total hip arthroplasty mechanical failures in a single academic institution not only related to modular implants (1), but also looking at demographics (2), underlying causes (i.e. BMI, sports and fracture pattern) (3), time after initial implantation (4), and implant choice as possible risk factors (5).

## Methodology

A retrospective trial was performed using the arthroplasty database at our academic center. Internal review board approval was obtained. All patients who presented to our outpatient clinic and the emergency department were included between 2011 and 2019. Radiographs and patient charts were searched for mechanical failures of total hip arthroplasty. Information on patients’ demographics, comorbidities, year of implant, hip arthroplasty survival, cemented or uncemented implantation, location of failure, mechanism of injury (high versus low energy), other implants used such as plate or cable wires for the treatment of periprosthetic fractures and subsequently revision surgery were all collected. Furthermore, information on the type of implanted arthroplasty was noted.

**Fig. 1:**
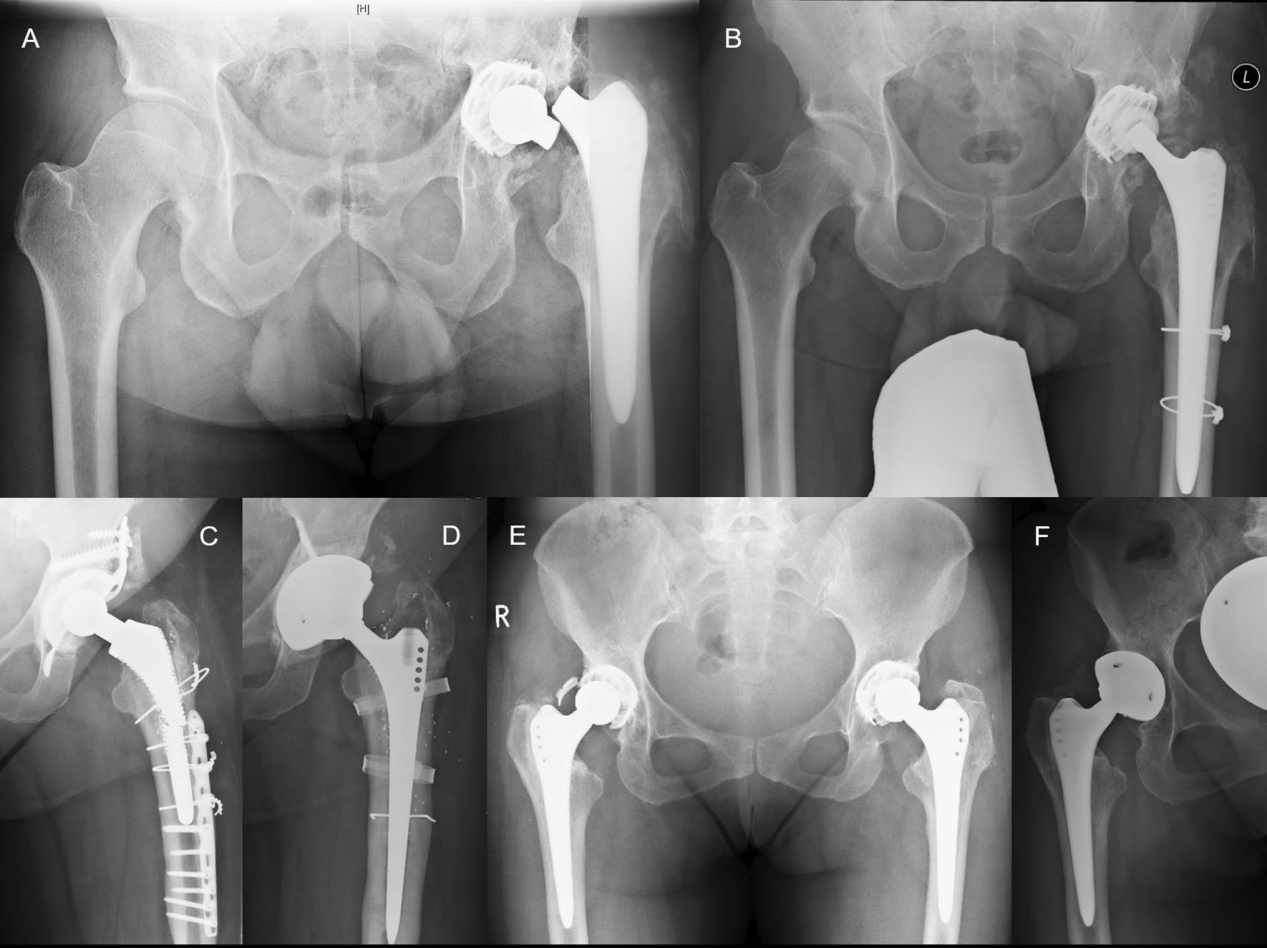
**A** Zimmer Alloclassic Zweymüller conus fracture, **B** after Stem exchange and replacement with a S&N SLR stem, **C** broken ESKA stem before and after THA exchange—Modular TMT cup and Zweymüller Revision stem **D**, **E** broken Zimmer Alloclassic cup and after Cup exchange (Zimmer TMT cup, **F**)

Hip arthroplasty failures were divided into cup versus femoral shaft. Additionally, the cup was subdivided into inlay versus cup failure, whereas breakage of the femoral shaft was divided into head, neck, shoulder or shaft fractures as illustrated in Fig. 1.

For statistical analysis, Microsoft Excel (Version 16.36) and IBM SPSS version 22 (IBM, Armonk, New York, USA) was used. Demographic data was analyzed for sex, and age in the hip arthroplasty failure groups. Shapiro-Wil testing was applied to identify whether variables were normally distributed. If normality was met, mean and standard error of the mean were calculated. The Pearson correlation coefficient was used to determine the association between the presence of infection, trauma, surgical approach, age at time of initial implantation, time elapsed between initial surgery and implant failure. The correlation coefficient is reported as *r* (between + 1 and − 1) to measure the strength and direction of a linear relationship. The level of significances was set to *p value ≤ 0.05; **p value ≤ 0.01; and ***p value ≤ 0.005.

In total, 13 total hip arthroplasty failures were observed. Female gender comprised of 61.5% (*n* = 8/13) of the THA group. This accounts for 0.9% of all revision arthroplasties (0.9%; *n* = 13/1421) performed during the period of interest. Overall, the mean age of this cohort was 69.08 ± 11.69 years at the time of the arthroplasty failure and the mean body mass index was 31.42 ± 5.29 kg/m^2^. Time elapsed between primary implantation and implant failure was 133.85 ± 103.12 months.

Arterial hypertension was noted in ten patients and obesity class I (BMI between 30 and 35 kg/m^2^) in five patients who suffered from THA failure. Other comorbidities include apoplexia, diabetes mellitus, poliomyelitis, myocardial infarction, epilepsy and cardiac insufficiency.

## Results

A structural implant failure was observed in most cases. The implant fractures at the femoral neck (conus) in four patients, at the stem in five cases, and at the inlay in one patient. In this same cohort, there are also two cup failures and one conus dislocation. In one cup failure, the broken cup cage (Zimmer Biomet, Warsaw, IN, USA) grew Cutibacterium acnes. In the other, the broken Alloclassic cup (Zimmer Biomet, Warsaw, IN, USA) grew Cutibacterium acnes and Staphylococcus epidermidis (*r* = − 0.021; *p* = 0.951). Two of these failures resulted from a fall at home when moving a heavy object. In the remaining patients, sudden onset of hip pain without any trauma was described (*r* = − 0.177; *p* = 0.625). Surgical approach chosen for revision was anterolateral (Watson–Jones) in all cases except for one that was posterolateral (*r* = − 0.065; *p* = 0.841). In this cohort, no clear underlying causes such as age, physical load or level of activity were identified to result in implant failure. The mean age was 69.08 ± 11.69 years (− 0.056; *p* = 0.862) with a time elapsed between primary implantation and implant failure of 133.85 ± 103.12 months (*r* = 0.179; *p* = 0.578). All patients denied high level of activity. Although the mean BMI was 31.42 ± 5.29 kg/m^2^ and seven patients were of obesity class I (five patients) or II (two patients), no correlation between the type of implant failure and obesity class was observed (*r* = − 0.531; *p* = 0.186).Revision surgery was indicated in all patients. Femoral stem and head exchange were performed in three patients. Additionally, inlay exchange in two further cases was required. In one case, a complete reconstruction using a plate and cerclage was performed. In those patients with cup failure, cup and head exchange was performed. For those with hip dislocation, an open reduction was executed and offset increased. In patients with a broken stem, revision focused on bypassing the fracture with a longer stem, supplemented by additional fixation such as cerclage wires as needed. Postoperatively, two complications were identified including one periprosthetic joint infection and the other is periprosthetic fracture. In both cases, further surgeries were required. However, no recurrent implant failure was observed. All cases are illustrated in Table 1.

**Table 1:** Listings of all individual total hip arthroplasty failures, comorbidities and subsequent treatment

Number	Age (years)	Gender	Side	Time between implantation and failure (months)	Level of energey	Implant	Previous surgery	Implant failure	Revision surgery	Diabetes mellitus/obesity	Infection (MSIS)
1	79	F	R	114	Low	Unclear	No	Modular Conus	Stem exchange S&N MIA stem 5, Ana-Nova Inlay 50, S&N Biolox Ceramic 32/L head	Yes/Yes	No
2	48	M	R	94	Low	Plasmacup SC, Excia stem, Modular Head, System sc	No	Conus	Stem exchange S&N SLR Stem 5, S&N Biolox Delta Ceramik 36/XL head	No/No	No
3	68	M	R	12	High	Likely DePuy Synthes Corail stem, TMT modular pressfit Cup 66, PE Inlay, 66/36, Metal head 36/XL	Revision arthroplasty after PJI and hip THA dislocation	Conus	Stem exchange S&N SLR Plus Stem 7, S&N Biolox Delta 36/L Ceramic head	No/No	No
4	52	M	L	111	Low	Zimmer Alloclassic Zweymüller, Zimmer Durasul Alloclassic CSF 28/55, Zimmer Durasul CoCr head 28/ + 4 long	No	Conus	Stem exchange S&N SLR stem, Zimmer Alloclassic 55/28, S&N Biolox Delta 28/L head	No/No	No
5	70	M	R	41	Low	Zimmer Revitan modular stem, cemented Durasul Inlay 48, metal head 36/L	Two stage revision arthroplasty after infection	Stem	Stem exchange, transfemoral osteotomy, Zimmer, Revitan modular stem (distal 18/200 mm, straight, proximal 75 mm, Metal head 36/M	No/No	No
6	75	F	L	70	Low	ESKA stem, cup cage with screw fixation, metal head	Two stage revision arthroplasty after infection and periprosthetic fracture	Stem	THA exchange Zimmer, Modular TMT Cup 58, Longevity-PE Inlay 32 mm, 2 screws, S&N Zweymüller Revision Stem 1, metal head 32/XL	No/No	No
7	83	F	L	287	High	Link Lubinus Classic Plus, Cemented cup, Cerclage greater Trochanter		Stem	Stem exchange, ORIF, LCP Plate 13 holes, Link cemented stem 30cmx135°, Zimmer metal head 32/XL	No/Yes	No
8	75	F	L	312	Low	Uncemented Judet total hip arthroplasty	Inlay exchange after PE bearing	Stem	Externally	No/No	No
9	78	F	R	181	Low	Likely Hermsdorf total hip arthroplasty	No	Inlay	Cup exchange S&N TMT Pressfit Revision 62 with screw fixation, cemented PE Cup 54, metal head 32/L, Impaction bone grafting	No/No	No
10	69	F	L	174	Low	Zimmer Burch Schneider Reinforcement ring, Zimmer SL Plus Stem	No	Cup	Cup exchange S&N Reko-Ring 44, cemented Cup 44, Impaction Bone grafting	No/Yes	No
11	65	F	L	0	Low	S&N SLR-stem 1, 28 mm/XL head, S&N Reko-Ring 44, cemented Müller II PE cup 42/28 mm,	Revision arthroplasty after infection	Head dislocation	Debridment and open reduction	No	No
12	51	F	R	53	Low	Zimmer Alloclassic Zweymüller stem, Zimmer Alloclassic cup	No	Cup	Cup exchange Zimmer, TMT cup 46, Longevity-PE Inlay 32, metal head 32/M for 12/14 mm conus, allogene spongiosa	No/No	Yes, P. acnes and Staph. epidermidis
13	85	M	R	291	Low	Zimmer Alloclassic Zweymüller, S&N Bicon Plus cup size 5	No	Stem	Stem exchange, transfemoral osteotomy, Zimmer, Revitan modular stem (distal 16/140 mm, straight, proximal 95 mm, Ceramic head 36/L, PE Inlay	No/No	No

## Discussion

In total, 13 mechanical failures were found. They affected the stem (*n* = 5), the neck (*n* = 4), followed by the cup (*n* = 2) and inlay. One patient sustained dislocation of the femoral head. No significant correlations between the implant failure and age at time of arthroplasty failure, BMI, physical load, level or energy, time elapsed between primary implantation and implant failure was observed.

Existing literature suggest that inlay is at risk for wear or failure [13]. In Germany, all implant failures are summarized in an annual report to be approximately 2.0% [14]. This is much higher rate than in our patient cohort at 0.4% (*n* = 13/3430). In this same cohort, we reported 1421 revision arthroplasties at a rate of 0.9% (*n *= 13/1421). [14] Although implant failure is a rare complication, numerous numbers have been reported to the Food and Drug Administration’s Manufacturer and User Facility Device Experience Database (MAUDE) [15]. Although not statistically significant, one risk factor for mechanical failure found in our study is obesity. Chee et al. found an increased general complication rate of 22% for BMI greater than or equal to 30 kg/m^2^ as compared to 5% in patients with BMI less than 30 kg/m^2^.[16] Similar findings were found by Haynes et al., who performed a systematic review noting a direct correlation between body mass index and complication rate. [17] In addition, implant manufacturers warn of increased complication rate in obese patients, although there is no strict weight limit [9]. There has been no correlation found between implant failure and sports participation in literature to date [10, 11]. Another potential risk factors is the usage of high frequency electrocautery, which can cause a change in the structure of the metal in implants and reduce fatigue strength. [18, 19]

The implants are either made of titanium alloy or cobalt-chromium. Latter ones have higher strength, although some reports of mechanical failure have been described. [20, 21] Improvement in material science and implant design have decreased complications rate since total hip arthroplasty was first introduced. To improve the fatigue strength of Ti6AI4V standard alloy, surface treatments like shot peening, deep rolling, ultrasonic shot peening and laser shock peening have been applied [5]. Since the taper is at risk in revision arthroplasty, two-step treatment of cut wire peening and glass bead blasting are especially critical to remove steel contamination. [22] Removal of residual ferrous particles on the surface minimized adverse mechanical and biological reaction. Furthermore, corundum grit and sand blasting are recommend to reduce endurance limit by 35–40% as compared to polished samples [23, 24]. Of course, over-peening can reduce mechanical strength by softening of the shot peened material [5].

Lateralized neck segment or extra long heads are predisposed to failure in a modular tapered design because of the higher bending moment generated at the modular junction. [15] Additionally, patients with BMI of ≥ 30 kg/m^2^ are at risk for revision surgery, which may increase steadily in the upcoming year [12]. To lower the risk, monoblock fluted tapered stems are recommended by some authors even in revision hip arthroplasty. For cup failures, uncoverage of the cup resulting from loss of bone stock such as hips dysplasia can be managed with screw-threaded cups, although this too can concentrates the stress on the implant.

In our cohort, four patients suffered from fractured conus (monoblock tapered prosthesis). In five patients, stem failures were observed, following one broken inlay, two cup failures and one conus dislocation. The mean time between implantation and failure was 133.85 ± 103.12 months. Females consisted of 61.5% (*n = *8/12) of the cohort. One patient suffered from infection with *P*. *acnes* and *S*. *epidermidis* (*n = *1/13; 7.7%), leading to septic loosening and ultimately mechanical failure. In most cases, a sudden atraumatic onset of pain was described but there was a history of falling in two patients. No correlation to physical load like endurance sports was observed. The mean BMI of the cohort was 31.42 ± 5.29 kg/m^2^. As such, most patients in this cohort qualified as obesity class I.

There are several limitations to this retrospective study. First, no comparative control group was included and we only found 13 total hip arthroplasty mechanical failures. Additionally, this is a single academic center study that may have an element of selection bias, since it is routine for our center to accept higher acuity patients from smaller hospitals. Additionally, although all failed implants were reported and sent to the manufacturer, we could not obtain a report as to the cause of failure mechanistically.

## Conclusion

Mechanical failure in total hip arthroplasties is rare and especially affects the stem and neck followed by the cup and inlay. No significant correlations between the implant failure, age, physical load, level of energy, time elapsed between primary implantation and implant failure was observed. Although no significant correlation to BMI was observed most patients qualified as obesity class I. For revision surgery the implant removal—especially the stem when broken—remains challenging and may require an ETO and special instruments for extraction.

## Appendix

### Technique for implant exchange

In patients suffering from stem or conus failure, explanting the remaining stem is difficult. To exclude infection a hip aspiration is recommended and the neocapsule should be sent for microbiological analysis.

In revising THA with conus failures, special extractor and high speed milling machines may be needed. Furthermore, conus failure can be revise with an innovative instrument that contains a *U* shaped adaptor with two coaxial holes on its jaws. This allows a hole to be drilled in a transverse direction in the proximal part of the remaining prosthesis, such that an adaptor can be attached by a holding pin [16]. Otherwise, the stem needs to be removed in total. Narrow osteotome, electronic bone mill, and stylus touch drill (i.e. Midas Rex, Medtronic, Medtronic, Fort Worth, USA) are all tools to disrupt bone-implant interface to allow for extraction. In implants with coating to promote osteo-integration, a transfermoral approach (i.e. Sarcophagus approach developed by Doré) or an extended trochanteric osteotomy (ETO) is suggested to release the prosthesis [17]. Although some authors suggest a controlled back-slap for stem extraction, we do not favor this method.

To repair the cortex cerclages either before or after stem re-implantation with or without strut allograft for augmentation could be considered. The authors suggest the use of a long stem bypassing the osteotomy site by at least two times the diameter of the femur.

For cup exchange an acetabular osteotome system (i.e. Explant Acetabular Cup Removal System, Zimmer Biomet, Warsaw, USA) can be used to expedite extraction for press fit cups. Alternatively, curved mallet or osteotome can be used also. In all cases, inlay and head exchanges are recommended. After surgery, antibiotics should be prescribed until all microbiological results are received. Full weight bearing is indicated in patients who underwent either only cup or short stem exchange. Patients who underwent ETO are recommended to be partial weight bearing.
